# Supplementary material for: Efficient Delivery of Hydrophilic Small Molecules to Retinal Cell Lines Using Gel Core-Containing Solid Lipid Nanoparticles
Source: Pharmaceutics. 2021 Dec 28;14(1):74. doi: 10.3390/pharmaceutics14010074 (PMC8780956; doi:10.3390/pharmaceutics14010074)
Supplement: Supplementary file 1 [file pharmaceutics-14-00074-s001.zip › pharmaceutics-1494569-supplementary.pdf]

pharmaceutics

Supplementary Materials: Efficient Delivery of Hydrophilic Small Molecules to Retinal Cell Lines Using Gel Core-Containing Solid Lipid Nanoparticles

Li Huang, Erico Himawan, Soumaya Belhadj, Raúl Oswaldo Pérez García, François Paquet Durand, Nicolaas Schipper, Matej Buzgo, Aiva Simaite and Valeria Marigo

Table S1. Formulation code and component mass dissolved in O-phase for each formulation.

Drug Delivery System (DDS)	Tripalmitin (GTP; mg)	Soy-Bean Lecithin (LCT; mg)	Stearic Acid (SA; mg)	Poly-ε-caprolactone (PCL; mg)	50/50 DL-lactide/glycolide (PLGA; mg)	Core
SLN.01	15	15	1	-	-	Aqueous
SLN.02	15	15	1	10	-	Aqueous
SLN.03	15	15	1	-	10	Aqueous
SLN.04	15	15	1	-	-	Gel Core
SLN.05	15	15	1	10	-	Gel Core
SLN.06	15	15	1	-	10	Gel Core

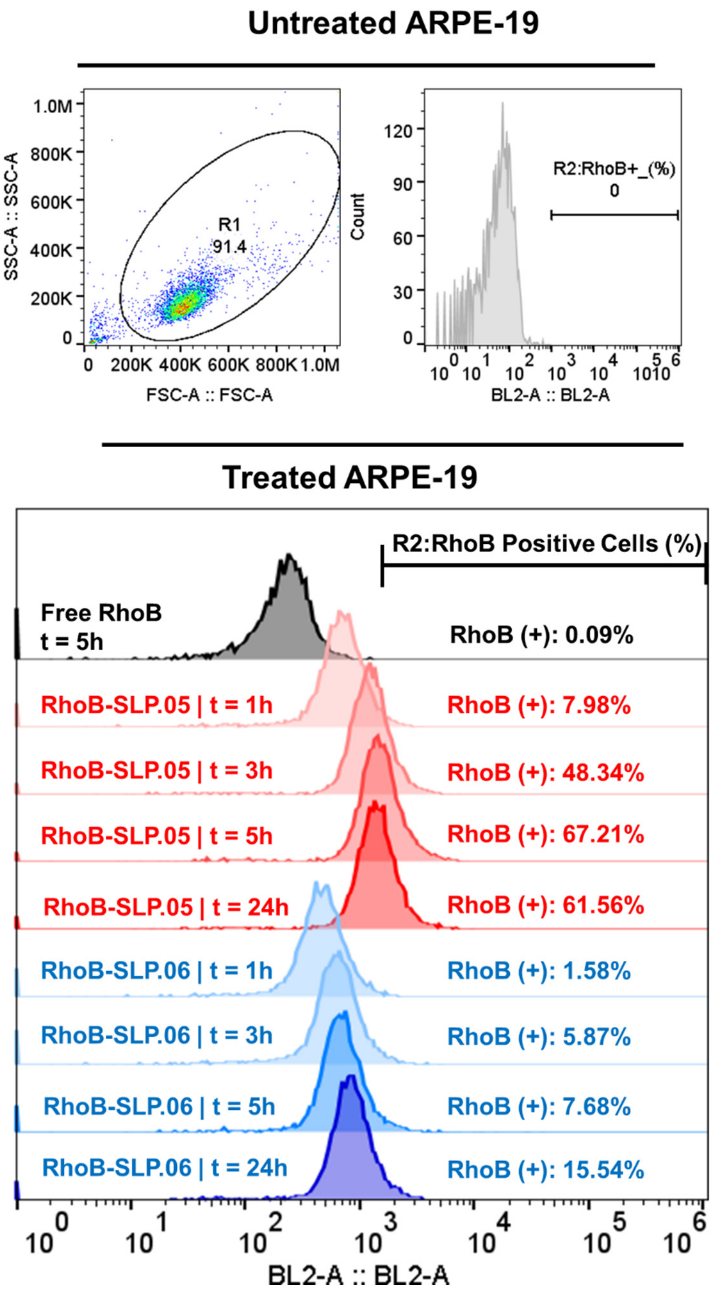

Figure S1. Flow cytometry analysis of ARPE-19 cells positive for RhoB after incubation with 200 μg/mL RhoB-SLN.05 or RhoB/SLN.06 at different time points. (**A**) Gating on ARPE-19 cells to determine RhoB positive cells. R1: gating for selecting cellular population; R2: gating to select RhoB+ cells based on blue laser (BL2-A) detector; FSC-A: forward scattering channel; SSC-A: side scattering channel. (**B**) Histogram overlay of RhoB relative fluorescence intensity and percentage of RhoB positive cells (RhoB(+)).
